# The Interactive Effects of Chilling, Photoperiod, and Forcing Temperature on Flowering Phenology of Temperate Woody Plants

**DOI:** 10.3389/fpls.2020.00443

**Published:** 2020-04-16

**Authors:** Huanjiong Wang, Hui Wang, Quansheng Ge, Junhu Dai

**Affiliations:** Key Laboratory of Land Surface Pattern and Simulation, Institute of Geographic Sciences and Natural Resources Research, Chinese Academy of Sciences, Beijing, China

**Keywords:** phenology, climate change, chilling, photoperiod, forcing, first flowering date, climate chamber experiment

## Abstract

The effects of winter chilling, spring forcing temperature, and photoperiod on spring phenology are well known for many European and North American species, but the environmental cues that regulate the spring phenology of East Asian species have not yet been thoroughly investigated. Here, we conducted a growth chamber experiment to test the effects of chilling (controlled by different lengths of exposure to natural chilling conditions), forcing temperature (12, 15, or 18°C) and photoperiod (14 or 10 h) on first flowering date (FFD) of six woody species (three shrubs and three trees) native to East Asia. The three-way analysis of variance (ANOVA) separately for each species showed that the effects of chilling and forcing temperature were significant for almost all species (*P* < 0.05). Averaged over all chilling and photoperiod treatments, the number of days until FFD decreased by 2.3–36.1 days when the forcing temperature increased by 3°C. More chilling days reduced the time to FFD by 0.7–26 days, when averaged over forcing and photoperiod treatments. A longer photoperiod could advance the FFD by 1.0–5.6 days, on average, but its effect was only significant for two species (including one tree and one shrub). The effects of forcing temperature and photoperiod interacted with chilling for half of the studied species, being stronger in the low chilling than high chilling treatment. These results could be explained by the theory and model of growing degree-days (GDD). Increased exposure to chilling coupled to a longer photoperiod reduced the GDD requirement for FFD, especially when plants grew under low chilling conditions. However, shrubs (except *Viburnum dilatatum*) had lower chilling and heat requirements than trees, suggesting that, by leafing out sooner, they engage in a more opportunistic life strategy to maximize their growing season, especially before canopy closure from trees' foliage. Our results confirmed the varying effects of these three cues on the flowering phenology of woody species native to East Asia. In future climate change scenarios, spring warming is likely to advance the spring phenology of those woody species, although the reduced chilling and shorter photoperiod may partly offset this spring warming effect.

## Introduction

The changed timing of recurring biological events becomes a global concern against the background of climate warming. The earlier spring phenophases (*e.g.*, budburst date, leaf-out date) and later autumn phenophases (*e.g.*, leaf coloring date) of woody plants were observed over the past several decades in middle and high latitudes of the Northern Hemisphere (Chmielewski and Rötzer, 2001; Menzel et al., 2006; Gonsamo et al., 2013; Ge et al., 2015; Templ et al., 2017). Such climate-associated phenological change could influence carbon assimilation by modifying the length of the growing season (Keenan et al., 2014; Xia et al., 2015). Differing rates of change in phenology among interacting species result in phenological mismatches between trophic levels (*e.g.*, prey and predator, plant and their pollinators), which affect biotic interactions and community structure (Peñuelas and Filella, 2001; Burkle et al., 2013; Choi et al., 2019; Damien and Tougeron, 2019). Plant phenology also had a feedback effect on climate systems by altering the biophysical attributes of the planet's terrestrial surface and atmospheric structure and composition (Richardson et al., 2013). Therefore, in order to adequately predict the future dynamics in vegetation–climate systems and their modeling, it is essential to understand the driving factors of plant phenology.

In temperate regions, temperature is the main factor determining the budburst date of woody plants (Sparks and Menzel, 2002; Walther et al., 2002). The experimental evidence amassed to date shows that temperature exerts various influences on spring phenology in different developmental stages, and the temperature cues could be divided into winter chilling and spring forcing (Cannell and Smith, 1983; Heide, 1993; Körner and Basler, 2010; Campoy et al., 2011; Hänninen et al., 2019). Many studies found that when twigs and saplings (with dormant buds) were exposed to a longer period of chilling temperatures in natural or controlled conditions, they needed less time to budburst under the same growth-promoting conditions (Pletsers et al., 2015; Man et al., 2017; Nanninga et al., 2017). This effect could be described as the negative relationship between chilling accumulation and heat requirements of plants (Cannell and Smith, 1983). In other words, a decrease in the amount of chilling during winter could increase the demand for cumulative forcing temperatures in spring. Compared with chilling, the forcing temperature during spring is a more recognized factor driving spring phenology. Presuming the rate of plant development is positively related to temperature, higher spring temperatures would lead to faster forcing-temperature accumulation and thus an accelerated budburst (Murray et al., 1989; Hänninen, 1990). Therefore, climate warming can exert dual effects on spring phenology, because the amount of chilling may decrease due to winter warming effects (Fu et al., 2015b). Compared with chilling, the impact of spring warming seems stronger, since the empirical evidence shows an earlier onset of spring phenological events has been broadly observed across Europe (Penuelas et al., 2002; Menzel et al., 2006), North America (Cayan et al., 2001; Miller-Rushing and Primack, 2008) and East Asia (Matsumoto et al., 2003; Ho et al., 2006; Ge et al., 2015; Wang et al., 2019b).

In addition to chilling and forcing temperature, the effect of photoperiod on spring phenology has recently attracted considerable attention (Way and Montgomery, 2015). To examine whether a species is sensitive to photoperiod relied so far on controlled experiments. If the twigs or saplings of a specific species exposed to a long photoperiod need less time to attain budburst than those under a short photoperiod, this species may be designated as a photoperiod-sensitive one (Basler and Körner, 2012). According to recent work, only a small number of woody species are sensitive to photoperiod (Zohner et al., 2016), and this photoperiod sensitivity is not related to species' traits such as successional niche (early *vs.* late-successional), xylem anatomy (diffuse or ring-porous xylem) and leaf persistence (evergreen or deciduous) (Way and Montgomery, 2015). Those plant species native to low latitudes are more likely to rely on spring photoperiod as a leaf-out cue (Zohner et al., 2016). Even for the photoperiod sensitive species, many studies suggest their budburst dates only respond to photoperiod under the condition of insufficient chilling (Laube et al., 2014; Zohner et al., 2016).

As discussed above, the spring phenology of woody plants depends on at least three interacting environmental cues. But when using long-term observational data, it is difficult to identify the effect of a specific cue, because all environmental cues will covary year by year except for photoperiod (Flynn and Wolkovich, 2018). Therefore, controlled experiments offer the best way to investigate the interactive effects of chilling, photoperiod, and forcing temperature on the spring phenology of woody plants. Because controlling the environment of mature trees or shrubs in the field is logistically difficult (and very costly), most of the previous studies have carried such experiments on dormant twigs of woody plants (Primack et al., 2015). In this approach, twig cuttings are brought indoors and placed in controlled conditions, such as growth chambers, where they are monitored until they leaf out or reach the phenological stage of interest. This method has proven to be useful and realistic, since no significant differences were detected in the timing of budburst between cuttings and donor trees for three typical tree species growing under the same climatic condition (Vitasse and Basler, 2014). Currently, twig experiments focusing on one or multiple cues have been applied to woody plants in North America (Nanninga et al., 2017; Flynn and Wolkovich, 2018) and Europe (Caffarra et al., 2011; Zohner et al., 2016). In Asia, although one study did assess the effect of forcing temperature on budburst date (Wang et al., 2019a), no multispecies study has yet evaluated all three major cues through the controlled experiment approach.

To begin filling this knowledge gap, this study focused on six woody plants originating from East Asia, to investigate how different chilling, forcing temperature, and photoperiod treatments vary in their effects on spring phenology. We aimed to test two hypotheses: (1) A higher forcing temperature and increased exposure to chilling accelerate spring phenology; (2) The impact of photoperiod on spring phenology is species-specific and is dependent on the chilling conditions.

## Materials and Methods

### Experimental Set-Up

Woody plant materials for the experiment were collected from Olympic Forest Park, Beijing (40° 01′ N, 116° 23′ E, 40–50 m above sea level), located 12 km north from the city center. Beijing has a typical continental monsoon climate (**Figure S1** in Data Sheet 2). Its summer months (June–August) are hot and rainy, with a mean temperature of 25.8°C and total precipitation of 364.5 mm (averaged from 1981 to 2010). Winter months (previous December to February) are cold and dry, having a mean temperature of –1.3°C and total precipitation of 9.2 mm. According to *a priori* criteria that the object plants should be widely distributed and native to East Asia, with high ornamental value, we selected six deciduous broadleaf woody plants for investigation (**Table 1**): three shrubs (winter jasmine, golden-bell, linden arrowwood) and three trees (Yoshino cherry, lilytree, wild peach). For each species, we selected three individuals as the parent plants from which to obtain the twig cuttings. Because our sampling location was a man-made forest, all the parent plants were of the same age (*ca.* 10 years old in 2017).

**Table 1:** Characteristics of the six studied plant species.

Common name	Scientific name	Family	Life form
Winter jasmine	*Jasminum nudiflorum*	Oleaceae	Deciduous broadleaved shrub
Golden-bell	*Forsythia suspensa*	Oleaceae	Deciduous broadleaved shrub
Linden arrowwood	*Viburnum dilatatum*	Caprifoliaceae	Deciduous broadleaved shrub
Lilytree	*Yulania denudata*	Magnoliaceae	Deciduous broadleaved tree
Yoshino cherry	*Cerasus yedoensis*	Rosaceae	Deciduous broadleaved tree
Wild peach	*Amygdalus davidiana*	Rosaceae	Deciduous broadleaved tree

**Figure 1:**
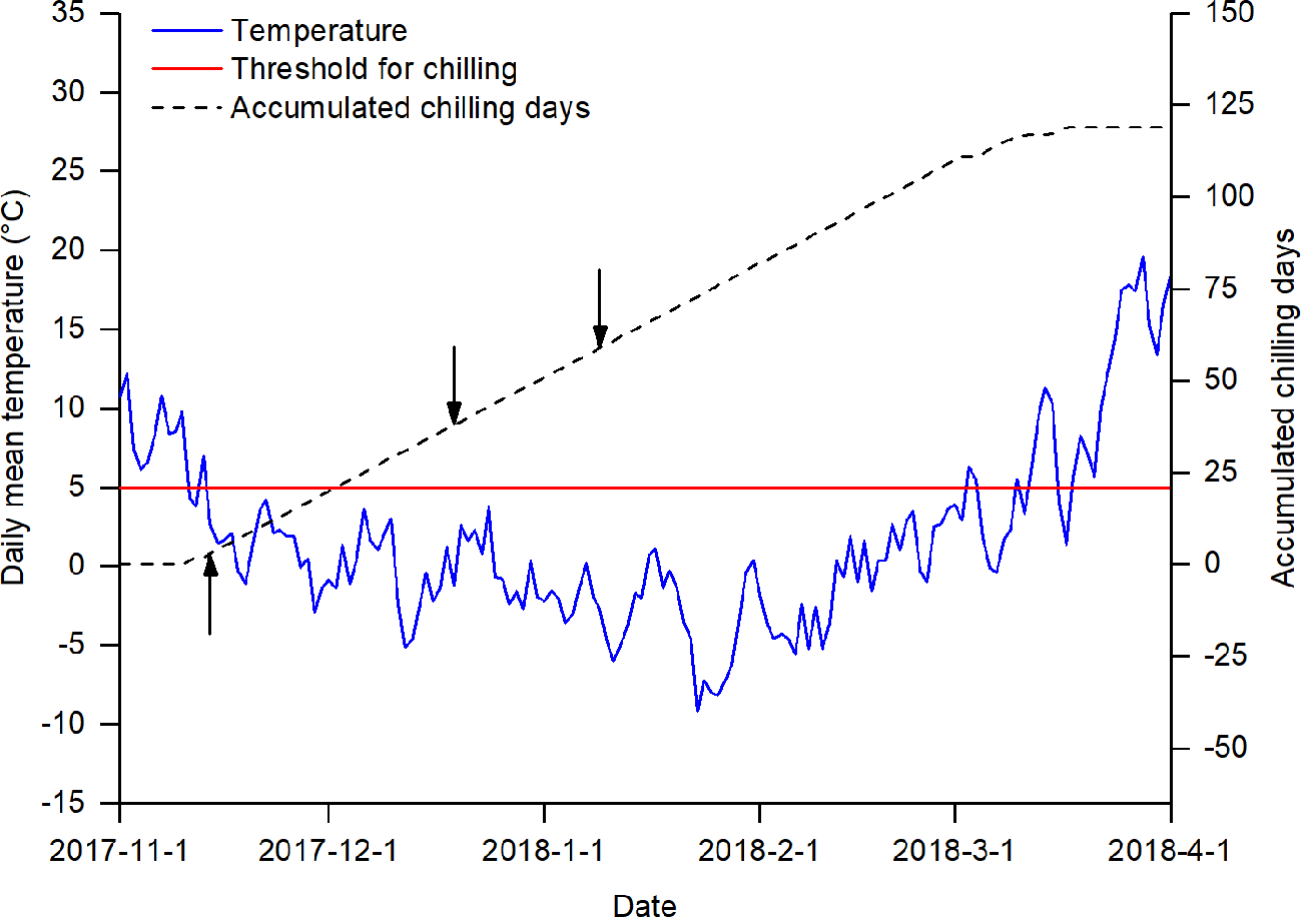
Daily mean temperature and accumulated chilling days from 1 November 2017 to 1 April 2018. The arrows indicate the three sampling dates. The red line indicates the threshold for accumulating chilling days.

The chilling treatments were imposed by manipulating the duration of plants' exposure to natural chilling conditions (**Figure 1**). According to previous studies (Cannell and Smith, 1983; Laube et al., 2014; Asse et al., 2018), a temperature below 5°C was generally effective for breaking dormancy. In Beijing, the daily mean temperature usually drops to 5°C in mid-November and to 0° in mid-December, reaching its lowest level in early January (**Figure S1** in Data Sheet 2). Therefore, in the winter season of 2017, we collected twigs of the studied species on three sampling dates (14 November 2017, 19 December 2017, and 9 January 2018). The amount of chilling at the sampling dates was measured by the number of chilling days, defined as the number of days when the daily temperature was below 5°C.

(1) $$CD(\text{t})=\sum_{t=t_{0}}^{t_{\text{s}}}1\;\text{if}\;T_{i}\leq 5$$

where, *CD* is the number of chilling days; *t*_0_ is the start date for chilling accumulation; *t*_0_ was set to 1 November, following its use in previous studies (Cannell and Smith, 1983; Laube et al., 2014); *t_s_* is the sampling date; and *T_t_* is the daily mean temperature (in °C) on day *t*. The daily mean temperature data for calculating the amount of chilling was obtained from the China Meteorological Data Service Center (http://data.cma.cn/site/index.html). Based on equation (1), three chilling treatments were achieved by collecting twigs at different times: low chilling (3 chilling days), intermediate chilling (38 chilling days), and high chilling (59 chilling days).

For each species, biological replicates were used in the form of one twig collected separately from three individuals. Thus, from each species on each sampling date, 18 twigs were cut (6 treatments × 3 individuals). To ensure all twigs had at least five flower buds we cut the twigs to variable lengths (usually 20–30 cm) as needed, since the cuttings' length had to be increase to accommodate those twigs with sparse buds. After cutting them, all the twigs were brought to the laboratory immediately, where they were placed into 0.25-L glass bottles filled with tap water. For each species, the three twigs from its different individuals were placed in one bottle, and six bottles were moved into different growth chambers (GXZ-500, Ningbo Jiangnan Instrument Factory, China).

In our experiment, the temperature treatments were designed to match the natural temperature variation in spring. According to Beijing's climate (**Figure S1** in Data Sheet 2), daily mean temperature generally increases from 12°C in early April to 18°C in late April. Thus, the three temperature treatments were set respectively to 12, 15, and 18°C.

In Beijing, the longest and shortest daylength within a year is 14.9 h (on the summer solstice) and 9.2 h (on the winter solstice), respectively. Thus, we chose two photoperiod treatments that approximated the maximum and minimum daylengths found under natural conditions. The long photoperiod treatment was 14 h (corresponding to the daylength in early May), while the short photoperiod treatment was 10 h (corresponding to the daylength in late January).

The treatments of photoperiod, chilling, and forcing temperature are summarized in **Figure S2** in Data Sheet 2. The growth chambers—in which the illumination, cooling, and heating systems were installed—could control the temperature and light duration automatically, according to user-defined settings. The lighting used in the growth chambers came from light-emitting diode (LED) tubes, in the form of cool white light with a photon flux density of 100 μmol m^−2^ s^−1^ (Red : Far Red = 3). To prevent the twigs and buds from drying out and failing to reach budburst, we put water-filled dishes with tissue paper into the chambers and maintained their relative humidity conditions above 70%. The twigs were re-cut and their water supply changed every second week to avoid blockages in their xylem caused by any fungi growing at the stem base.

Observations were made three times per week, for five months. For each twig, the date when at least one fresh flower had opened (sometimes several flowers opened simultaneously) was recorded as the first flowering date (FFD), which corresponded to BBCH 60 (Meier, 2001). For each species, the FFD was defined as the average of the FFD of three twigs.

### Statistical Analysis

The response variable, *i.e.*, the number of days from sampling date to FFD, was analyzed in relation to three explanatory factors (categorical variables): (1) chilling days in natural conditions; (2) forcing temperature in the growth chambers, and (3) daylength in the growth chambers. For each species, the experimental data (see the **Supplementary Data Sheet**) were analyzed using a general linear model (GLM) to do a three-way analysis of variance (ANOVA) in SPSS 16.0 software (IBM Corp, Armonk, NY, USA). The mean values between treatments (or their combinations) were compared using Fisher's LSD test, at an alpha significance of 0.05.

To explain the impact of chilling accumulation and photoperiod on heat requirements of FFD, the growing degree-days (GDD) requirement was calculated as the accumulated degree-days from the sampling date to FFD on daily basis (equation 2).

(2) $$GDD=\sum_{t=t_{\text{s}}}^{t_{\text{f}}}\left(T_{\text{g}}\left(\text{t}\right)-T_{b}\right)$$

where, *t_s_* is the sampling date; *t*_f_ is the FFD; *T_g_*(t) is the forcing temperature in the growth chamber (in °C), at day *t*; and *T_b_* is the threshold temperature for heat accumulation. Considering that 5°C was the threshold above which biological activity was generally believed to start (Cannell and Smith, 1983; Fu et al., 2015a), *T_b_* was set to 5°C in this study.

## Results

### Effects of Forcing Temperature on First Flowering Date

**Figure 2:**
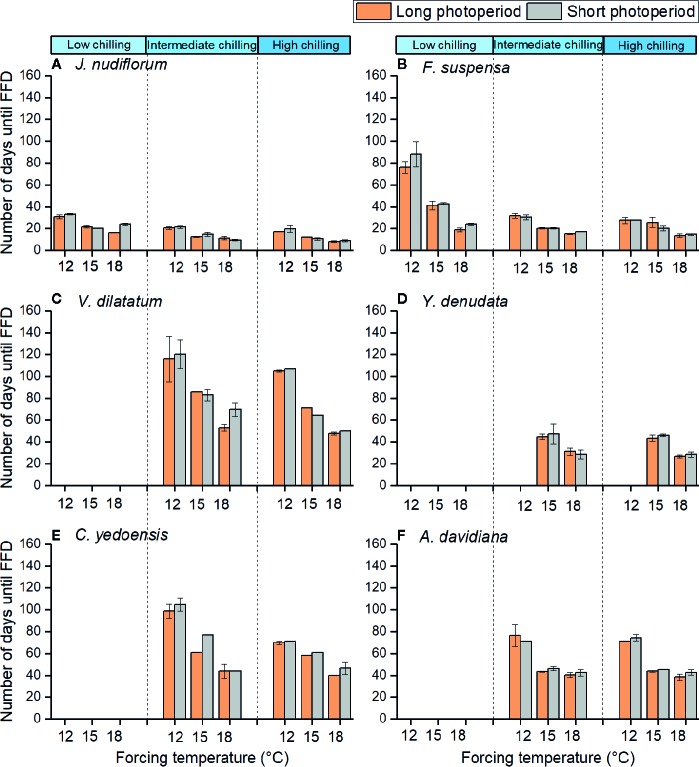
Number of days from sampling dates to the first flowering date of six species at different chilling, forcing temperature and photoperiod conditions. The low (3 chilling days), intermediate (38 chilling days), and high (59 chilling days) indicate different chilling treatments, respectively. 12, 15, and 18°C indicate different forcing temperatures. Different colors indicate long (14h) and short (10 h) photoperiods. The flowers of the last four species could not open in low chilling condition. The error bars indicate standard deviation among three replicates (no error bar is presented when the standard deviation equals to zero). **(A)** *J. nudiflorum*, **(B)** *F. suspensa*, **(C)** *V. dilatatum*, **(D)** *Y. denudata*, **(E)** *C. yedoensis*, **(F)** *A. davidiana*.

The impact of forcing temperature on the number of days until FFD was statistically significant (*P* < 0.01) for all species investigated (**Table 2**). The number of days until FFD occurred significantly decreased as the forcing temperature increased (**Figure 2**, **Table 3**). Averaged over all chilling (low, intermediate, and high chilling) and photoperiod (long and short photoperiod) treatments, the reduction in the number of days until FFD with increased temperature was greatest for *V. dilatatum*. The time to FFD was shortened by 57.1 days at a forcing temperature of 18°C compared with that of 12°C. Another responsive species was *C. yedoensis*, for which the difference in the number of days until FFD was 42.4 days between 12 and 18°C. Regarding *A. davidiana* and *F. suspensa*, compared with the forcing temperature of 12°C, their number of days until FFD at a forcing temperature of 18°C shortened by 32.3 days and 29.7 days on average, respectively. The flower(s) of *Y. denudata* could not open at 12°C, but at 18°C, the number of days required for FFD was 16.7 days less than that at 15°C. For *J. nudiflorum*, the effect of forcing temperature on its FFD was the weakest among the six species, as the difference between 12 and 18°C was just 10.8 days.

**Table 2:** Analysis of variance for the effects of forcing temperature (F), chilling (C), photoperiod (P), and their interactions on the number of days until the first flowering date.

Variables	*J. nudiflorum*	*F. suspensa*	*V. dilatatum*	*Y. denudata*	*C. yedoensis*	*A. davidiana*
F	397.0**	314.9**	174.9**	100.5**	416.2**	371.6**
P	16.41**	2.794	1.073	0.362	21.41**	1.470
C	459.4**	326.5**	30.11**	1.005	131.7**	0.398
F × P	5.734**	2.417	2.833	0.814	2.700	1.174
F × C	6.373**	88.77**	0.395	0.090	58.14**	0.031
P × C	5.620**	5.465**	1.928	0.492	2.475	1.979
F × C × P	13.35**	1.348	0.587	0.362	5.437*	1.626

The values shown are the F-statistic of the analysis of variance (ANOVA). **P < 0.01; *P < 0.05.

**Table 3:** Mean differences in the number of days until the first flowering date (FFD) among the different forcing, chilling, and photoperiod treatments.

Comparisons	*J. nudiflorum*	*F. suspensa*	*V. dilatatum*	*Y. denudata*	*C. yedoensis*	*A. davidiana*
**Forcing temperature**						
18–15°C	−2.3(−0.8)**	−11.2(−3.7)**	−21.0(−7.0)**	−16.7(−5.6)*	−20.7(−6.9)**	−3.6(−1.2)**
15–12°C	−8.5(−2.8)**	−18.5(−6.1)**	−36.1(−12.3)**	N	−21.8(−7.3)**	−28.7(−9.6)**
18–12°C	−10.8(−1.8)**	−29.7(−5.0)**	−57.1(−9.5)**	N	−42.4(−7.1)**	−32.3(−5.4)**
**Photoperiod**						
14h–10h	−1.3**	−1.6*	−2.6	−1.0	−5.6**	−1.4
**Chilling**						
3CD–38CD	9.2(0.26)**	26.0(0.74)**	N	N	N	N
38–59CD	2.3(0.11)**	0.8(0.04)	13.8(0.66)**	1.7(0.08)	13.8(0.66)**	0.7 (0.03)
3–59CD	11.6(0.21)**	26.8(0.48)**	N	N	N	N

For forcing temperature, the values in parentheses indicate the changes in the number of days until FFD per °C of warming. For chilling, the values in the parentheses indicate the changes in the number of days until FFD per chilling day (CD). The significance of multiple pairwise comparisons was determined with Fisher's LSD test **P < 0.01; *P < 0.05.

Furthermore, the response of FFD to forcing temperature was nonlinear (**Table 3**). At 12°C, the changed number of days until FFD per °C of warming (12 *vs.* 15°C) was stronger than that at 15°C (15 *vs.* 18°C) for all six studied species (**Table 3**). However, the extent of reduced temperature sensitivity of FFD varied among the species. The most obviously different was *A. davidiana*, for which the temperature sensitivity of FFD decreased from 9.6 days per °C warming (12 *vs.* 15°C) to 1.2 days per °C of warming (15 *vs.* 18°C). In stark contrast, the corresponding decreased temperature sensitivity was only 0.4 days/°C for *C. yedoensis*, being the weakest among the six species (**Table 3**).

### Effects of Chilling on First Flowering Date

Under the low chilling condition, only *J. nudiflorum* and *F. suspensa* could open flower normally, while the other four species could not under the forcing conditions (**Figure 2**), suggesting that the chilling requirements varied among species. For all species investigated, more chilling days lessened the required time to reach FFD, on average (**Table 3**), but this effect was only significant (*P* < 0.01) for four species (**Table 2**). Compared with low chilling, the intermediate and high chilling conditions led FFD occurring 9.2 and 11.6 days earlier in *J. nudiflorum* and 26.0 and 26.8 days earlier for *F. suspensa*, respectively. Concerning *V. dilatatum* and *C. yedoensis*, they required 13.8 days less until their FFD in the high chilling than intermediate chilling treatment. By contrast, for *Y. denudata* and *A. davidiana*, the difference in the number of days until FFD between intermediate chilling and high chilling conditions was small (respectively 1.7 and 0.7 days), and not significant.

Further, in the low chilling condition (3 chilling days), a one chilling-day increase could lead to 0.26- and 0.74-day advance in the FFD of *J. nudiflorum* and *F. suspensa*, respectively, but their corresponding FFD response decreased to 0.11 and 0.04 days per chilling day under intermediate chilling (38 chilling days). Thus, for these two species, if the amount of chilling was high enough, the effect of further chilling became weak. A similar phenomenon also occurred in *Y. denudata* and *A. davidiana*. Yet, in the intermediate chilling condition, further chilling was also effective for hastening FFD in *V. dilatatum* and *C. yedoensis* (**Table 3**).

### Effects of Photoperiod on First Flowering Date

Averaged over all forcing and chilling treatments, the number of days until FFD under the long photoperiod was 1.0 to 5.6 days less than that under a short photoperiod (**Table 3**). Thus, a long photoperiod could promote the FFD of all six studied species, but such an effect of photoperiod was only significant (*P* < 0.01) for *J. nudiflorum* and *C. yedoensis* (**Table 2**). For *J. nudiflorum*, under the forcing temperature of 18°C coupled to a low chilling condition, a long photoperiod shortened the time required for its FFD by 8 days when compared to a short photoperiod. Long photoperiod treatment caused *C. yedoensis* to flower 16 days earlier than did a short photoperiod under the forcing temperature of 15°C and intermediate chilling condition.

### Interaction Effect of Forcing Temperature, Chilling, and Photoperiod

The interaction between forcing temperature and chilling was significant (*P* < 0.01) for *J. nudiflorum*, *F. suspensa*, and *C. yedoensis* (**Table 2**). **Figure 2** shows that under the same amplitude of warming, the time to FFD of these species decreased more in low and intermediate chilling conditions than in a high chilling condition. Hence, the temperature sensitivity of FFD was stronger with lower chilling.

Another noteworthy interaction effect was that between chilling and photoperiod (**Table 2**), which was significant (*P* < 0.01) for two species (*J. nudiflorum* and *F. suspensa*). A sooner FFD under the long photoperiod was observed more frequently in low and intermediate chilling conditions than in the high chilling condition. Although other interaction effects (forcing temperature × photoperiod, forcing temperature × chilling × photoperiod) were significant in one or two species, no consistent pattern was evident.

### Relationship Between Heat Requirement, Chilling, and Photoperiod

**Figure 3:**
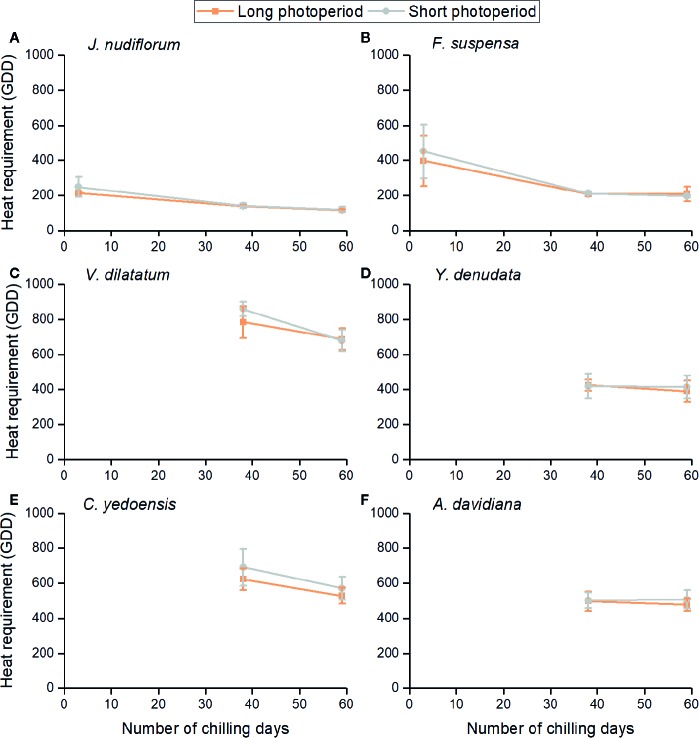
Relationships between mean heat requirement of the first flowering date (FFD) of six species and chilling accumulation under long and short photoperiod conditions. Heat requirement was calculated as the accumulated degree days (above 5°C) from the sampling date to FFD. The error bars indicate standard deviation among 3 temperature treatments × 3 replicates. **(A)** *J. nudiflorum*, **(B)** *F. suspensa*, **(C)** *V. dilatatum*, **(D)** *Y. denudata*, **(E)** *C. yedoensis*, **(F)** *A. davidiana*.

The impacts of three environmental cues could be described by the relationship between GDD requirements and chilling days under two photoperiods (**Figure 3**). The heat requirement for FFD clearly varied among species. The mean GDD requirement across all treatments was high for *V. dilatatum* (753.2) and *C. yedoensis* (603.7), followed by *A. davidiana* (496.4) and *Y. denudata* (412.0), and least in *F. suspensa* (279.2) and *J. nudiflorum* (163.3) whose GDD requirements were low. For the same chilling and photoperiod condition, the GDD requirement was similar among different forcing temperature treatments, although the difference among individuals still existed (see the error bar in **Figure 3**). Higher forcing temperatures would lead to GDD accumulating more quickly, which could explain the promoting effects of forcing temperature upon FFD. The increase in chilling days reduced the GDD requirement of all species, but this effect was apparently weak for both *Y. denudate* and *A. davidiana*. For two other species (*J. nudiflorum* and *F. suspensa*), whose flowers opened in the low chilling condition, their reduced GDD requirements were mitigated by more chilling days.

Furthermore, the GDD requirements of FFD were higher under the short than long photoperiod for most experimental treatments (**Figure 3**), but the difference in the mean GDD requirement between long and short photoperiods was not statistically significant for all chilling treatments and species (*t*-test, *P* > 0.05). We noticed that the effect of photoperiod was more pronounced in the low and intermediate than high chilling condition for *J. nudiflorum, F. suspensa,* and *V. dilatatum*. For the other species, no obvious difference in the effect of photoperiod was found among chilling treatments.

## Discussion

### Effects of Forcing Temperature

Our results indicate that increasing the forcing temperature significantly promotes the FFD of woody plants, which is consistent with findings on spring phenology of other species, such as leaf budburst in *Betula pubescens* (Caffarra et al., 2011), *Fagus sylvatica*, *Tilia cordata*, *Salix x smithiana* (Caffarra and Donnelly, 2011) and 28 woody species from two North American forests (Flynn and Wolkovich, 2018). In addition to experimental studies, the long-term observational data has also showed that interannual changes in spring phenology are negatively correlated with the temperature averaged from one to three months before these springtime events (Estrella et al., 2007; Szabó et al., 2016; Wang et al., 2018; Xu et al., 2018). Therefore, the advance in spring phenology observed over the past decades could be attributed to spring warming. Since multiple General Circulation Models (GCMs) predict the warming trends would continue under all Representative Concentration Pathway (RCP) scenarios, except RCP 2.6 (IPCC, 2013), spring phenology will likely continue to advance earlier in the future.

**Figure 4:**
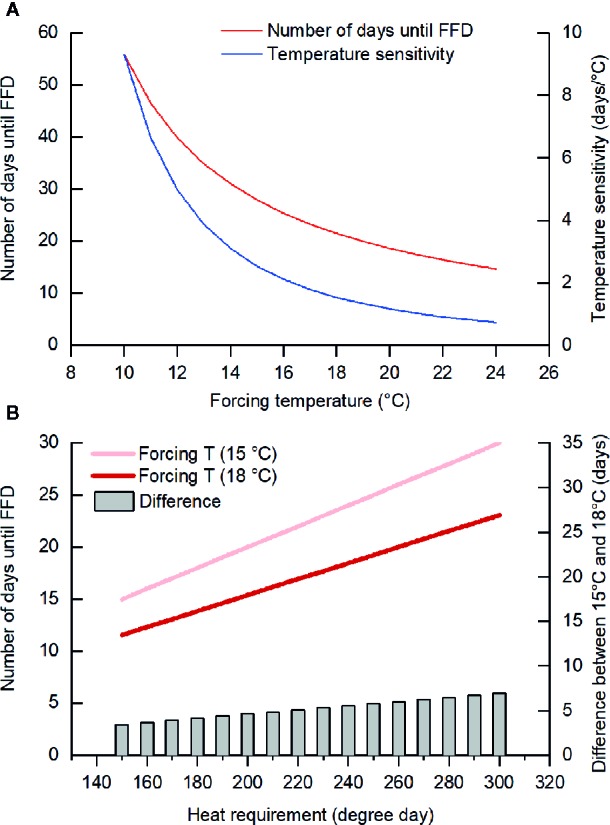
Number of days until the first flowering date (FFD) based on the heat requirement. **(A)** When the heat requirement is constant (279.2 degree days, the threshold temperature of 5°C), temperature sensitivity (changes in the number of days until FFD per °C warming) decreases with the increase in forcing temperature. **(B)** The difference in the number of days until FFD between forcing temperature of 15 and 18°C was larger when the heat requirement became higher (with lower chilling).

Furthermore, we found that the temperature sensitivity of FFD decreased as the forcing temperature was increased. Previous studies also reported similar results (Morin et al., 2010; Fu et al., 2013; Wang et al., 2019a). For example, the temperature sensitivity of leaf unfolding in oak and beech trees decreased drastically when warming exceeded +4°C (Fu et al., 2013). In our study, the mean GDD requirement for FFD in *F. suspensa* was 279.2 degree days (threshold of 5°C) and the number of days until FFD at a forcing temperature of *T* could thus be calculated as 279.2/(*T*–5); hence, with a higher *T*, the change in number of days to FFD per °C of warming decreases (**Figure 4A**). Accordingly, this reduced temperature sensitivity with greater forcing temperatures could be explained by the theory of GDD requirement. However, in our experiment, we did not consider the possible impact of temperature variation because we used a fixed (nonfluctuating) temperature treatment. In natural conditions, the temperature sensitivity of woody species' leaf and flowering phenology was weaker at locations with larger variance in local spring temperature (Wang et al., 2014) and higher occurrence frequency of freezing events (Wang Y. et al., 2019).

### Effects of Chilling

Our results suggest that increased exposure to chilling lessens the time to flowering and reduces the GDD requirements of the studied species. Such a chilling effect was also reported by other experimental studies done on leaf or flower budburst of other woody species (Okie and Blackburn, 2011; Vitasse and Basler, 2013; Pletsers et al., 2015; Man et al., 2017; Du et al., 2019). For example, in Ontario, Canada, the amount of heat required for leaf budburst of seven species decreased progressively with cumulative chilling hours (Man et al., 2017). In Ireland, a longer chilling duration resulted in earlier leaf budburst of *Betula pubescens* (German clone) and *Populus tremula* (Irish clone), and for both species less thermal time was needed to reach maximum budburst (Pletsers et al., 2015). Long-term observational data also confirms this assumption because the heat requirement of leaf-out for several European species has increased over the past 30 years, mainly due to the warming-related reductions in chilling (Fu et al., 2015a). In the model plant, hybrid aspen (*Populus tremula x tremuloides*), the physiological basis of its chilling-mediated control of bud break has been uncovered (Singh et al., 2018), and many genes and phytohormones are involved in this intricate process. Thus, the current body of evidence suggests the negative relationship between the heat requirement and amount of chilling is widespread among perennials. In natural conditions, several studies have also found that reductions in chilling days during the last several decades may have limited how much earlier leaf budburst could advance, to some extent (Fu et al., 2015b; Asse et al., 2018; Vitasse et al., 2018).

Nevertheless, the effect of chilling on FFD clearly varied among species. In the low chilling condition (three chilling days), only *J. nudiflorum* and *F. suspensa* could open their flowers normally, which suggests these species need none or very few chilling days to break their dormancy. Yet three chilling days could not break the dormancy of the other four species, since they failed to open their flowers even after a 5-month-long forcing treatment. In the intermediate chilling (38 days) condition, dormancy was released for all the six species, but the effect of further chilling was not consistent. The time to flowering for four species (*J. nudiflorum, F. suspensa*, *Y, denudata,* and *A. davidiana*) was very similar under intermediate and high chilling conditions. Therefore, in Beijing and other regions with cold winters, the amount of chilling is likely enough for breaking dormancy before mid-December, and the amount of chilling experienced by plants over the whole winter exceeded the chilling requirements of these species (Chmielewski and Götz, 2017). For them, the countervailing effect from reduced chilling on spring phenological advance was found to be weak. For other species (*V. dilatatum* and *C. yedoensis*), high chilling was also effective in advancing the time to flowering compared to their response to intermediate chilling. Thus, in natural conditions, for species whose spring phenology response was still sensitive to chilling in the high chilling state, the warming-related reduction in chilling days would delay the advancement of their spring phenological events.

### Effects of Photoperiod

In other reported experiments, a long (short) photoperiod usually refers to daylength longer than (shorter than) 12 h, such as 12 h *vs* 8 h (Flynn and Wolkovich, 2018) and 16 h *vs.* 8 h (Zohner et al., 2016). We compared the photoperiod effect between 14 and 10 h, finding that a long photoperiod could hasten the FFD of all species albeit to differing extent and it reduced the GDD requirement of FFD in most cases. Consistent with our results from Asia, for 28 woody species from two North American forests, long photoperiod caused a 5-day advance in leaf budburst compared with that induced by short photoperiod at the community level (Flynn and Wolkovich, 2018). However, in our study, the effect of photoperiod on FFD was only significant for two of the six investigated species. One recent study found that with low chilling, only 35% of 173 temperate species relied on spring photoperiod as a cue for leaf-out (Zohner et al., 2016). Similarly, the photoperiod had little effect on both leaf-out and flowering events in 37 subtropical woody species (Song et al., 2020). Therefore, it would seem that most woody plant species do not use photoperiod as an external regulator of spring phenology.

In our experiments, though the difference between photoperiod treatments reached 4 h of light a day, the long photoperiod only advanced the FFD earlier by 1.0 to 5.6 days, an effect weaker than from forcing temperature and chilling. However, for plants grown in field conditions, the daily change in photoperiod would be constant among years at a given location. Assuming one year has an earlier FFD (t_e_) and another year has a later FFD (t_l_), the photoperiod was the same before t_e,_ but the year with later FFD received additional sunshine hours in the timespan from t_e_ to t_l_. We believe that such a difference in photoperiod (t_e_ to t_l_) would be smaller than that of our photoperiod treatment lasting for five months. Therefore, the restriction of daylength on the advances in the spring phenology of woody plants was weak and difficult to detect under natural conditions.

### Interaction of Multiple Cues

The ANOVA demonstrated that forcing temperature and chilling interacted to affect three species (*J. nudiflorum*, *F. suspensa*, *C. yedoensis*). The effect of forcing temperature on FFD of these species was stronger in low and intermediate chilling conditions compared to high chilling condition. Taking *F. suspensa* as an example, we presumed its GDD requirements for FFD increased from 150 to 300 degree days with a decrease in the amount of chilling. Subsequently, for different GDD requirements, we could simulate the number of days until FFD occurs at forcing temperatures of 15 and 18°C. As **Figure 4B** shows, a higher GDD requirement (in the low chilling condition) leads to more pronounced difference in the elapsed time to FFD between 15 and 18°C. Thus, the interaction between forcing temperature and chilling upon FFD documented here could be explained by the theory of GDD requirement.

The effect of photoperiod and chilling on FFD interacted for two species (*J. nudiflorum* and *F. suspensa*). As shown in **Figure 3**, the heat requirement was higher under a long than short photoperiod treatment when twigs of these species had not fully chilled yet. Similar results were also found in other species such as *Fagus sylvatica* (Zohner and Renner, 2015; Fu et al., 2019). With low chilling, 35% of 173 species leafed out later under short-day conditions than under those of long-days, but only 2% of species responded sensitively to photoperiod when exposed to high chilling (Zohner et al., 2016). All these pieces of evidence together suggest that high chilling functions to reduce the photoperiod sensitivity of woody plants. Another assumption for a photoperiod effect is that a long photoperiod could compensate for too little chilling (Myking and Heide, 1995). However, the photoperiod *Betula pubescens* experienced during a chilling treatment did not affect its leaf budburst (Caffarra et al., 2011). In *Fagus sylvatica*, its leaf primordia only reacted to light cues late in dormancy after accumulating enough warm days (Zohner and Renner, 2015). Therefore, for photoperiod-sensitive species, a long photoperiod was likely to increase the effect of forcing temperature rather than act as a substitute for chilling's effect.

### Difference Among Plant Functional Types

As proposed by Körner and Basler (2010), because shrubs are shorter-lived and early successional plants, they may adopt a more risky life strategy to maximize their growing season when compared to trees (although not all tree species are long-lived, late-successional). Therefore, we expected shrubs to exhibit a lower chilling and heat requirement and to be less responsive to photoperiod. For chilling and forcing, our results generally support this hypothesis. In high chilling conditions, the mean GDD requirement—averaged over all forcing temperature and photoperiod treatments—for the FFD of *J. nudiflorum* and *F. suspensa* was only 118.1 and 203.0 degree days, respectively. Because *V. dilatatum* was the only species which flowered later than its timing of leaf-out in this study, the mean GDD requirement for its FFD was 683.9 degree days. If we consider this shrub's first spring event, the mean GDD requirement for the first leaf-out date of *V. dilatatum* was only 245.1 degree days. Relative to shrubs, the mean GDD requirement of three trees reached 402.1 to 550.2 degree days, which is higher than the GDD requirement for the first spring events of the three shrub species. Furthermore, with very low chilling (3 chilling days), shrubs (except *V. dilatatum*) could normally flower, but trees could not, suggesting the chilling requirement of shrubs was lower than trees in most cases. This difference in heat and chilling requirements between successional life-history strategies is consistent with the findings of Laube et al. (2014). However, the two species that responded significantly to photoperiod in our study consisted of one shrub and one tree; hence, our results do not support the hypothesis that tree species are more photosensitive than shrub species.

## Conclusions

In this study, we reported on a multispecies climate chamber experiment that tests the effects of forcing temperature, chilling, and photoperiod on the spring phenology of six woody species native to China. Increased forcing temperature, chilling, and daylength hastened the FFD of all species, although the effects of these cues varied and were not always significant for all species. Also, for several species, the impact of chilling interacted with forcing temperature and photoperiod, *i.e.*, the effect of forcing temperature and photoperiod tended to be stronger under a low chilling condition. These effects could be explained by the GDD theory, as more chilling exposure and a longer photoperiod reduced the GDD requirement to attain FFD. Because species responded in a complex way to multiple environmental cues, accurately predicting the FFD at the community level is challenging. In future climate change scenarios, spring warming would possibly advance the spring phenology of woody plants, but the reduced chilling and shorter photoperiod may limit the effect that spring warming has on those species having a high chilling requirement. According to our results, the impact of chilling and photoperiod on spring phenology is likely weaker than that from spring warming, because most of the woody plants are photoperiod-insensitive and the influence of chilling is generally weak at mid- to high-latitudes or at high elevations where the amount of chilling is already sufficient.

## Data Availability Statement

All datasets generated for this study are included in the **Supplementary Material**.

## Author Contributions

HuaW designed this experiment. HuaW and HuiW performed this experiment. HuiW analyzed the data. HuaW and HuiW wrote this manuscript. QG and JD extensively revised the writing.

## Funding

This work was funded by the National Key R&D Program of China (Grant No. 2018YFA0606103), National Natural Science Foundation of China (41871032), Youth Innovation Promotion Association, CAS (Grant No. 2018070), and Program for “Kezhen” Excellent Talents in IGSNRR, CAS (Grant No. 2018RC101).

## Conflict of Interest

The authors declare that the research was conducted in the absence of any commercial or financial relationships that could be construed as a potential conflict of interest.

## Supplementary Material

Data Sheet 1 Supplementary data sheet.

Data Sheet 2 Supplementary figure.
