# Supplementary material for: The Interactive Effects of Chilling, Photoperiod, and Forcing Temperature on Flowering Phenology of Temperate Woody Plants
Source: Front Plant Sci. 2020 Apr 16;11:443. doi: 10.3389/fpls.2020.00443 (PMC7176907; doi:10.3389/fpls.2020.00443)
Supplement: Data Sheet 2 — Supplementary figure. [file DataSheet_2.docx]

Supplementary Material





**Supplementary Figure S1.** The daily mean temperature and precipitation in Beijing averaged from 1981-2010.


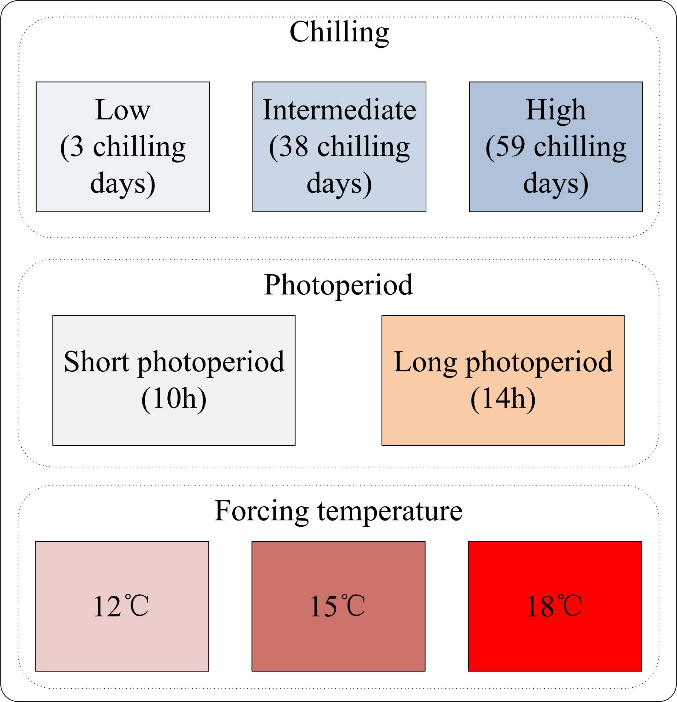


**Supplementary Figure S2.** The experimental design in this study
